# Effect of Nasal Obstruction on Continuous Positive Airway Pressure Treatment: Computational Fluid Dynamics Analyses

**DOI:** 10.1371/journal.pone.0150951

**Published:** 2016-03-04

**Authors:** Tadashi Wakayama, Masaaki Suzuki, Tadashi Tanuma

**Affiliations:** 1 Department of Otolaryngology, Teikyo University, Tokyo, Japan; 2 Department of Otolaryngology, Teikyo University Chiba Medical Center, Chiba, Japan; 3 Department of Applied Fluid Dynamics and Energy Machinery Systems, Joint Program Center, Teikyo University, Tokyo, Japan; The Chinese University of Hong Kong, HONG KONG

## Abstract

**Objective:**

Nasal obstruction is a common problem in continuous positive airway pressure (CPAP) therapy for obstructive sleep apnea and limits treatment compliance. The purpose of this study is to model the effects of nasal obstruction on airflow parameters under CPAP using computational fluid dynamics (CFD), and to clarify quantitatively the relation between airflow velocity and pressure loss coefficient in subjects with and without nasal obstruction.

**Methods:**

We conducted an observational cross-sectional study of 16 Japanese adult subjects, of whom 9 had nasal obstruction and 7 did not (control group). Three-dimensional reconstructed models of the nasal cavity and nasopharynx with a CPAP mask fitted to the nostrils were created from each subject’s CT scans. The digital models were meshed with tetrahedral cells and stereolithography formats were created. CPAP airflow simulations were conducted using CFD software. Airflow streamlines and velocity contours in the nasal cavities and nasopharynx were compared between groups. Simulation models were confirmed to agree with actual measurements of nasal flow rate and with pressure and flow rate in the CPAP machine.

**Results:**

Under 10 cmH_2_O CPAP, average maximum airflow velocity during inspiration was 17.6 ± 5.6 m/s in the nasal obstruction group but only 11.8 ± 1.4 m/s in the control group. The average pressure drop in the nasopharynx relative to inlet static pressure was 2.44 ± 1.41 cmH_2_O in the nasal obstruction group but only 1.17 ± 0.29 cmH_2_O in the control group. The nasal obstruction and control groups were clearly separated by a velocity threshold of 13.5 m/s, and pressure loss coefficient threshold of approximately 10.0. In contrast, there was no significant difference in expiratory pressure in the nasopharynx between the groups.

**Conclusion:**

This is the first CFD analysis of the effect of nasal obstruction on CPAP treatment. A strong correlation between the inspiratory pressure loss coefficient and maximum airflow velocity was found.

## Introduction

Obstructive sleep apnea (OSA) is caused by repeated reduction or cessation of breathing due to narrowing or occlusion of the upper airway during sleep. Nasal continuous positive airway pressure (CPAP) is currently the non-invasive treatment of choice for the majority of OSA patients. CPAP treatment markedly attenuates obstructive apnea, hypopnea, respiratory effort-related arousal, snoring, and flow limitation, and improves sleep fragmentation [1]. However, a major clinical trial found relatively low long-term adherence to nasal CPAP, from 40% to 80% depending on the usage metric employed [2]. Among complaints during nasal CPAP, nasal congestion is particularly prevalent and can significantly compromise compliance. Physicians commonly evaluate nasal obstruction by clinical assessments such as nasal resistance measurement, but there are few studies on the effects of nasal obstruction on airflow in CPAP treatment.

Computational fluid dynamics (CFD) analyses of three-dimensional (3D) airway models can be used for dynamic assessment to evaluate the real-time effects of narrowing on airway flow. CFD analyses based on the Navier–Stokes equations can predict 3D distributions of airflow velocity, volumetric flow rate, air pressure, turbulence measures, streamlines, and shear stress on the flow path walls.

To the best of our knowledge, no CFD analysis of the nasal cavity and nasopharynx under CPAP conditions has been reported. We have developed a biomechanical methodology involving CFD for analysis of nasal cavity and nasopharynx airflow. The purpose of this study was to clarify quantitatively the relation between airflow velocity and pressure loss coefficient (*Cp*) in subjects with and without nasal obstruction.

## Method

### Subjects

The Ethics Committee of Teikyo University approved this study (approval number 14–063) and written informed consent was obtained from all subjects. We conducted an observational cross-sectional study of 16 Japanese adults with suspected OSA who were examined at our ENT outpatient clinic. On the basis of nasal endoscopy, CT findings, and nasal resistance measured with an anterior rhinomanometer, subjects were classified according to the presence or absence of nasal obstruction. Patients with nasal obstruction (nasal obstruction group) had nasal diseases such as septal deviation, nasal allergy, or nasal polyp and total nasal resistance ≥ 0.41 Pa/cm^3^/s [mean age 44.7 ± 12.7 years, range 26−65 years; body mass index (BMI) 25.2 ± 3.2 kg/m^2^, range 19.1–29.4 kg/m^2^; n = 9 (8 men and 1 woman)]. Patients without nasal obstruction (control group) did not have such nasal diseases or complain of nasal obstruction and had total nasal resistance ≤ 0.18 Pa/cm^3^/s [mean age 51.4 ± 18.5 years, range 25−71 years; BMI 26.5 ± 3.2 kg/m^2^ range 23.3 − 29.7 kg/m^2^; n = 7 (3 men and 4 women)]. Nasal resistance was measured with an anterior rhinomanometer (HI-801, Chest M.I., Inc., Tokyo, Japan) in the supine position 1 h before sleep studies. Left, right, and total inspiratory nasal resistance at negative 100 Pa was measured. The nasal resistance values (0.41 and 0.18 Pa/cm^3^/s) were derived from the mean ± 2 standard deviations in a Japanese population [3]. Exclusion criteria were (i) pulmonary disease such as asthma or chronic obstructive pulmonary disease and (ii) evidence of adenoid or tonsil hypertrophy. Overnight polysomnography was conducted according to our previous investigations of subjects with suspected OSA [4].

### CFD analyses and actual measurement

**Fig 1:**
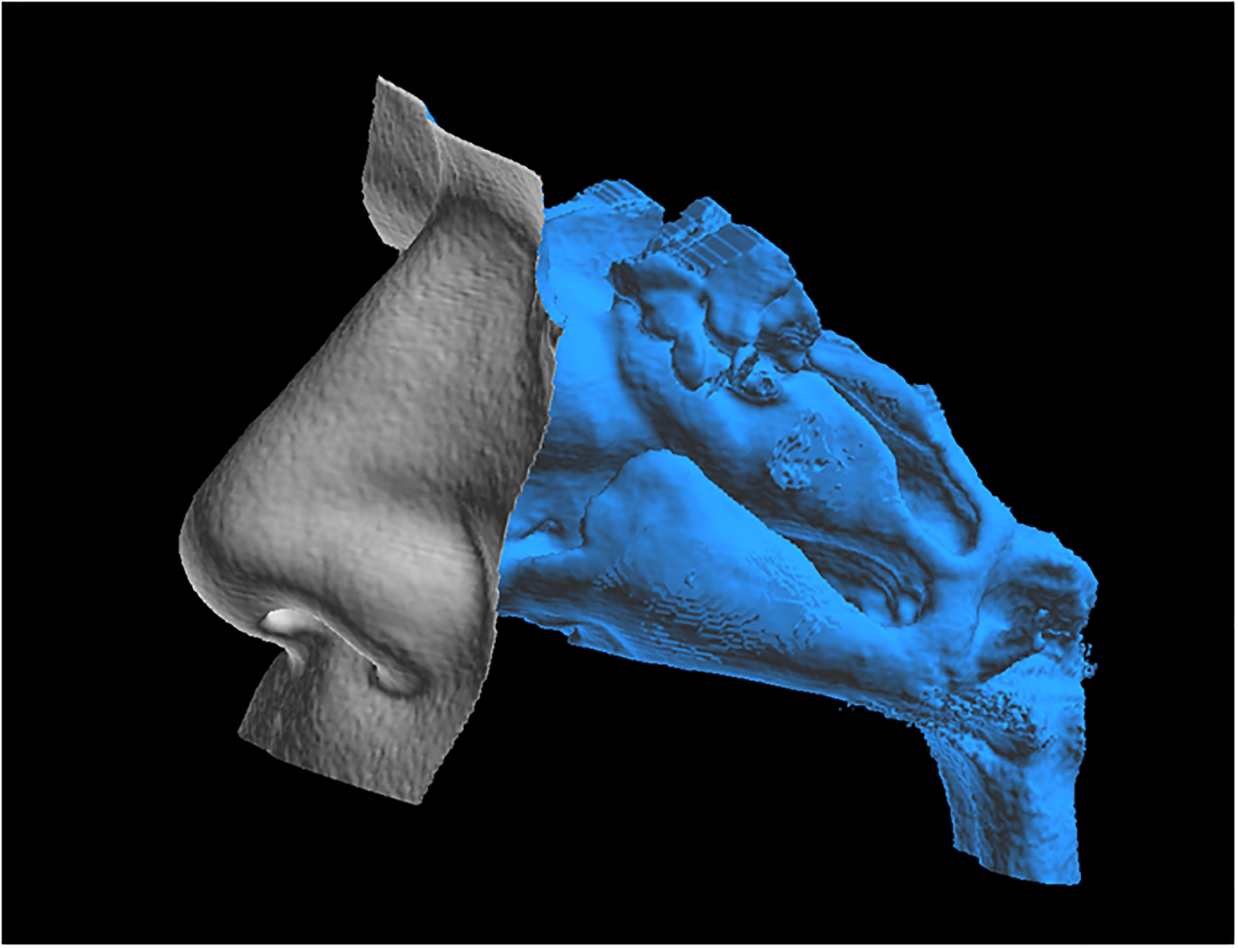
STL model of a nasal cavity and nasopharynx.

**Fig 2:**
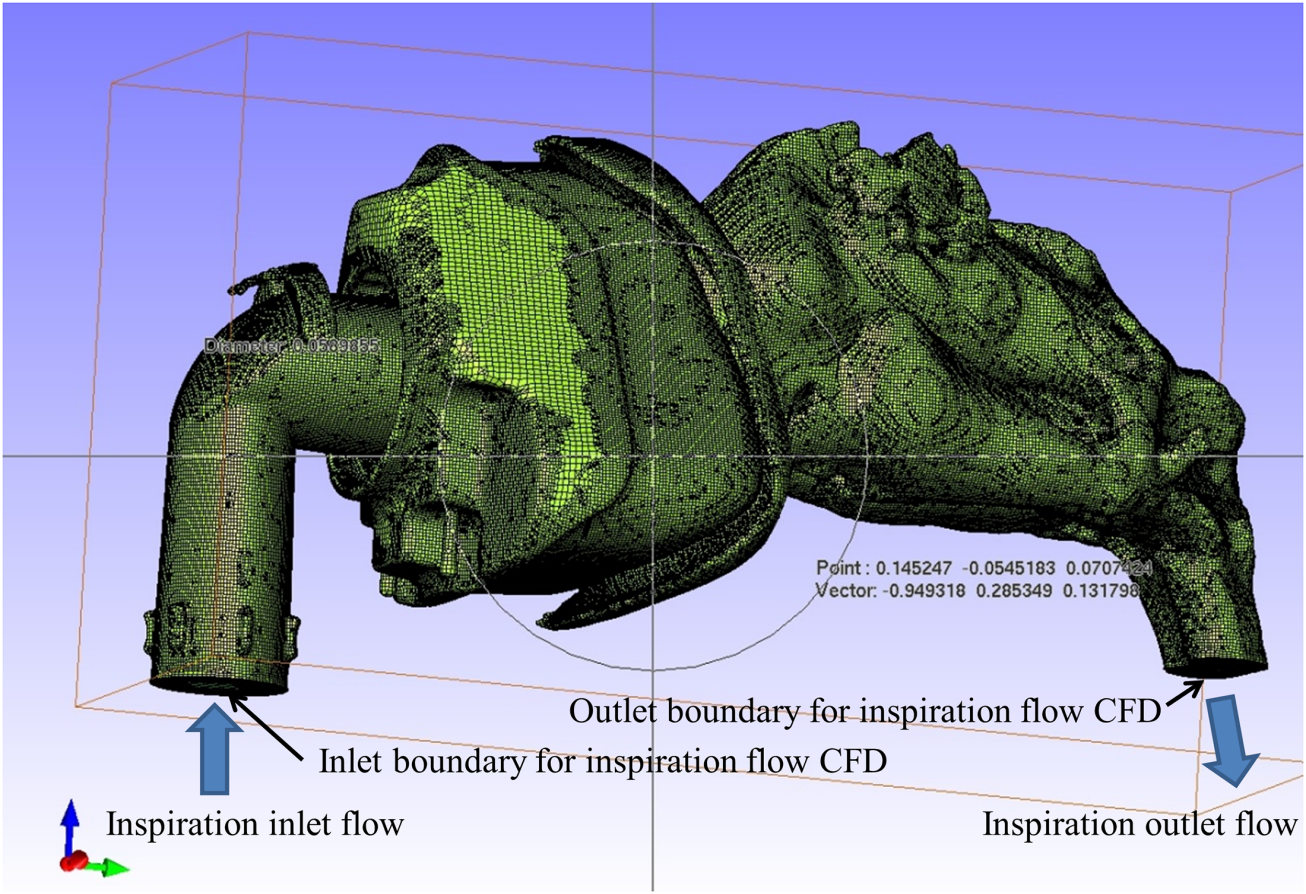
Digital unstructured grid models.

CT scans of the nose, sinuses, and nasopharynx were taken at 0.4-mm intervals and individual 3D reconstructed stereolithography (STL) models created using image analysis software (INTAGE Volume Editor, Cybernet Systems, Ann Arbor, MI). These 3D reconstructed STL models included the nasal cavity and nasopharynx, but excluded the paranasal sinuses and soft tissue surrounding the nasal cavity. The surfaces were highly corrugated due to artifacts of digitization and therefore were smoothed to facilitate computational meshing (Fig 1). Also included in the CPAP condition simulations was a model of a CPAP mask that fits to the nostrils. The geometry of the CPAP mask was measured using an industrial high-accuracy CT scanner (TOSCANER-32251μhd, Toshiba IT & Control Systems, Tokyo, Japan). STL geometry data of the nasal airway and STL geometry data of the measured CPAP mask were combined into a single set of geometry data to generate a CFD mesh. Fig 2 shows the CFD computational mesh for the flow path inside the CPAP mask, nasal cavity, and nasopharynx. For inspiratory flow CFD analysis, the inlet boundary was set at a cross section of the CPAP mask inlet pipe and an outlet boundary was set at a cross section of the nasopharynx (Fig 2). For expiratory flow CFD analysis, the inlet boundary and outlet boundary were interchanged. From our observational experiences of MRI reconstruction for a patient during CPAP, we confirmed that the dilatation of the walls in the nasal cavity and the nasopharynx is small enough to be regarded as solid under CPAP condition.

**Fig 3:**
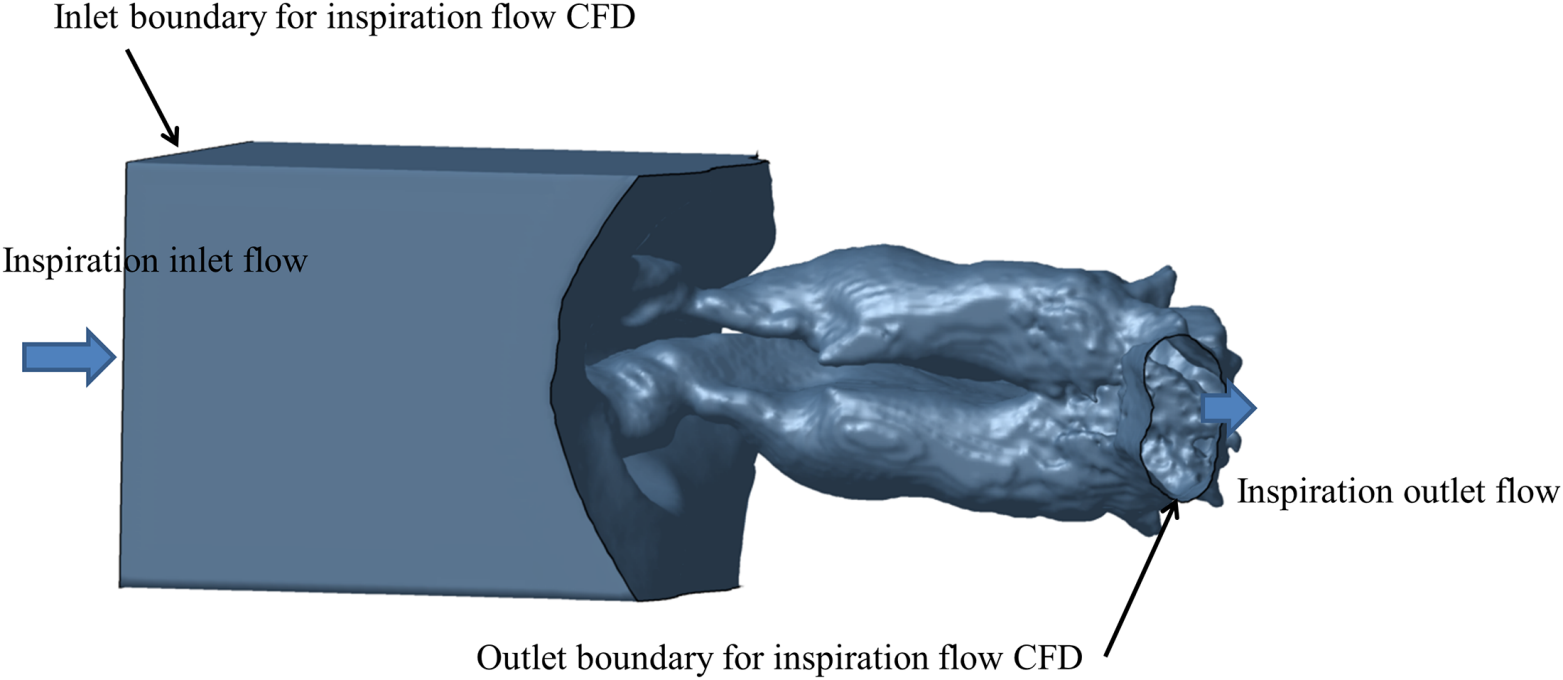
CFD computational domain of the nasal cavity, the nasopharynx, and a box-shaped closed space in front of the nostrils.

Fig 3 shows the CFD computational domain of the nasal cavity, the nasopharynx, and a box-shaped closed space in front of the nostrils without CPAP. A necessary and sufficient closed space was set in front of the nostrils to simulate natural inlet flow upstream the nostrils for the CFD simulation of the nasal airflow without the CPAP mask. For inspiratory flow CFD analysis, an inlet boundary was set at the cross section of the box-shaped closed space in front of the nostrils. Furthermore, the surrounding four plane surfaces were defined as slip solid surfaces, because these side solid surfaces are used only for limiting the CFD domain to prevent unnecessary calculations of very low-speed flow far from the nostrils. An outlet boundary was set at the cross section of the nasopharynx shown in Fig 3. For expiratory flow CFD analysis, the inlet boundary and outlet boundary were interchanged in the same way as in the CPAP analysis.

The digital unstructured grid models were meshed with 8 million hexahedral cells using the INTAGE Volume Editor and HEXPRESS^™^/Hybrid (NUMECA, Brussels, Belgium) (Fig 2). Three-dimensional geometrical modeling from medical image data and CFD analyses were conducted using a methodology described in our previous modeling studies of an artery with cerebral aneurysm [5,6]. For cases both with and without the CPAP mask, inlet boundary conditions were set with total pressures of CPAP (10 cmH_2_O) or atmospheric pressure conditions, and the inlet velocity distributions were approximated as flat neglecting the boundary layer. This was done because the flow path distance between the inlet boundaries and the flow path inside the nose were aerodynamically long enough and the effect of the inlet boundary layer was negligible with respect to flow in the nasal flow path. The outlet boundary conditions were set with static pressures that corresponded to the volume flow conditions for the current cases.

In our CFD analysis, surface roughness was neglected and all flow path surfaces were approximated as smooth but no-slip surfaces except for the four sidewalls surrounding inlet box-shaped closed space. For this reason, we assumed that the computed pressure resistance and the *Cp* would be slightly smaller than the actual nasal airflow. However, it was clear that the cross-sectional area of the nasal flow path changed rapidly, and flow separation occurred in many sections of the flow path. The pressure loss due to the flow separation was thus dominant and larger than the pressure loss due to surface friction. Consequently, the present CFD method can be used for relative comparisons between the nasal obstruction cases and the control cases.

The CFD calculations were separately carried out for inspiratory flow cases and expiratory flow cases. All calculations were steady-state calculations using the maximum instantaneous flow rates measured during inspiration and expiration.

The averaged Reynolds number under CPAP conditions in the present study was around 3500 that were larger than that of critical circular pipe (2300). Thus, we assumed that the inlet regions of the current flow paths with and without the CPAP mask were laminar flow. However, the flow velocity through very narrow sections might become high and some turbulent flow regions seemed to occur. Spalart-Allmaras one equation turbulence model was used with the extended wall function for all cases in the current CFD study, because the flow separations would play a leading role even in the current transition condition. The inlet turbulence boundary conditions were set with turbulence viscosity for 0.0001 m^2^/s in our empirical models.

The CFD convergences were determined with the assumption that the average residual of CFD iterations should be less than 10^-6 or the mass flow-rate difference between inlet and outlet boundaries should be less than 0.5%. We initiated current flow-path CFD calculations using a 3-million mesh grid point model and increased the number of grid points up to the current mesh density, 8-million grid points. However, the final calculation results did not significantly deviate from those obtained using the initial coarse mesh, suggesting that the current mesh grid points were sufficiently fine for our purposes.

Simulation models were confirmed to agree with measured airflow values. Actual measurements of nasal flow rate were performed using a Fleisch pneumotachometer (Laminar Flow Meter LFM-317^®^, Metabo, Lausanne, Switzerland), along with pressure and flow rate in the CPAP machine (XTAuto^®^, Apex, Taipei, Taiwan).

Under 10 cmH_2_O CPAP, the instantaneous maximum volumetric flow rate was 889 ml/s for inspiration and 300 ml/s for expiration. Without CPAP, the instantaneous maximum volumetric flow rate was 200 ml/s for inspiration and 200 ml/s for expiration. Simulations without CPAP were performed for the comparative study between nasal resistance of CFD simulations and that of actual measurements with rhinomanometry.

Airflow simulations were conducted using Navier–Stokes equations in CFD software (FINE^™^/Hexa ver. 2.10.4, NUMECA, Brussels, Belgium). Simulations were run over a 24-h period on a 64-bit workstation with 24 GB of memory and 6 CPUs.

For simulating CPAP conditions, atmospheric pressure (101325 Pa = 1033.26 cmH_2_O) plus 10 cmH_2_O was applied to the inlet of a CPAP mask. Air density (ρ) was 1.212 kg/m^3, and air mass flow rate was 899.0 *10^-6*1.212 = 10.90 * 10^–4 kg/s as the flow rate for inspiration. For non-CPAP conditions, atmospheric pressure at 20°C was applied to the inlet boundary shown in Fig 3 with a volumetric flow rate of 200 ml/s for inspiration and 200 ml/s for expiration. Air density was 1.204 kg/m^3 and air mass flow rate was 200.0 * 10^-6 * 1.204 = 2.408*10^-4 kg/s. Nasal wall boundary conditions were heat-insulated walls with viscosity and turbulence taken into consideration. A no-slip boundary condition was applied on all nasal airway surfaces.

The CPAPs are usually equipped with air leakage systems. Air leakage in CPAP is mainly through the slots around the inlet pipe of the CPAP mask. We measured the actual leakage flow rate, then, the total flow rate through the right and left cross-sectional areas at the nostril was calculated for both inspiratory and expiratory conditions. The CFD model of inlet pipe was simplified by closing the leakage slots, however, leakage effects were included in our CFD analyses.

We used definite values of volumetric flow rate, inlet and outlet boundary conditions, and constant geometry of CPAP mask for all cases to compare the effects of nasal obstruction accurately.

The inspiratory pressure loss coefficient defined with nose inlet air way dynamic pressure (*Cp)* was calculated from inlet volume flow (m^3^/s), cross-sectional area at the nostrils (m^2^) as measured by CT, air velocity at the nostrils (*V*_*in*_), inlet pressure at the nostrils (*P*_*in*_), and outlet pressure at the nasopharynx (*P*_*out*_) as shown in Eqs (1) and (2).

$$Vin=\frac{\text{inlet volume flow}}{\text{cross}-\text{sectonal area at the nostrils}}$$ (1)

$$Cp=\frac{\left(Pin-Pout\right)}{\frac{\rho {\left(Vin\right)}^{2}}{2}}$$ (2)

*Cp* was nondimentional parameter. All cross-sectional areas at the nostril were measured using the CT three dimensional geometry data in all present cases.

### Statistical analysis

All descriptive statistics for all variables are presented as the mean ± standard deviation. Differences between unpaired subjects were evaluated by the Mann–Whitney U test. A *p* value < 0.05 was considered statistically significant. Correlations between parameters were analyzed using Spearman’s correlation coefficient. All statistical analyses were performed using the Statistical Package for Social Science, version 11.01 (SPSS Inc., Chicago, IL).

## Results

**Fig 4:**
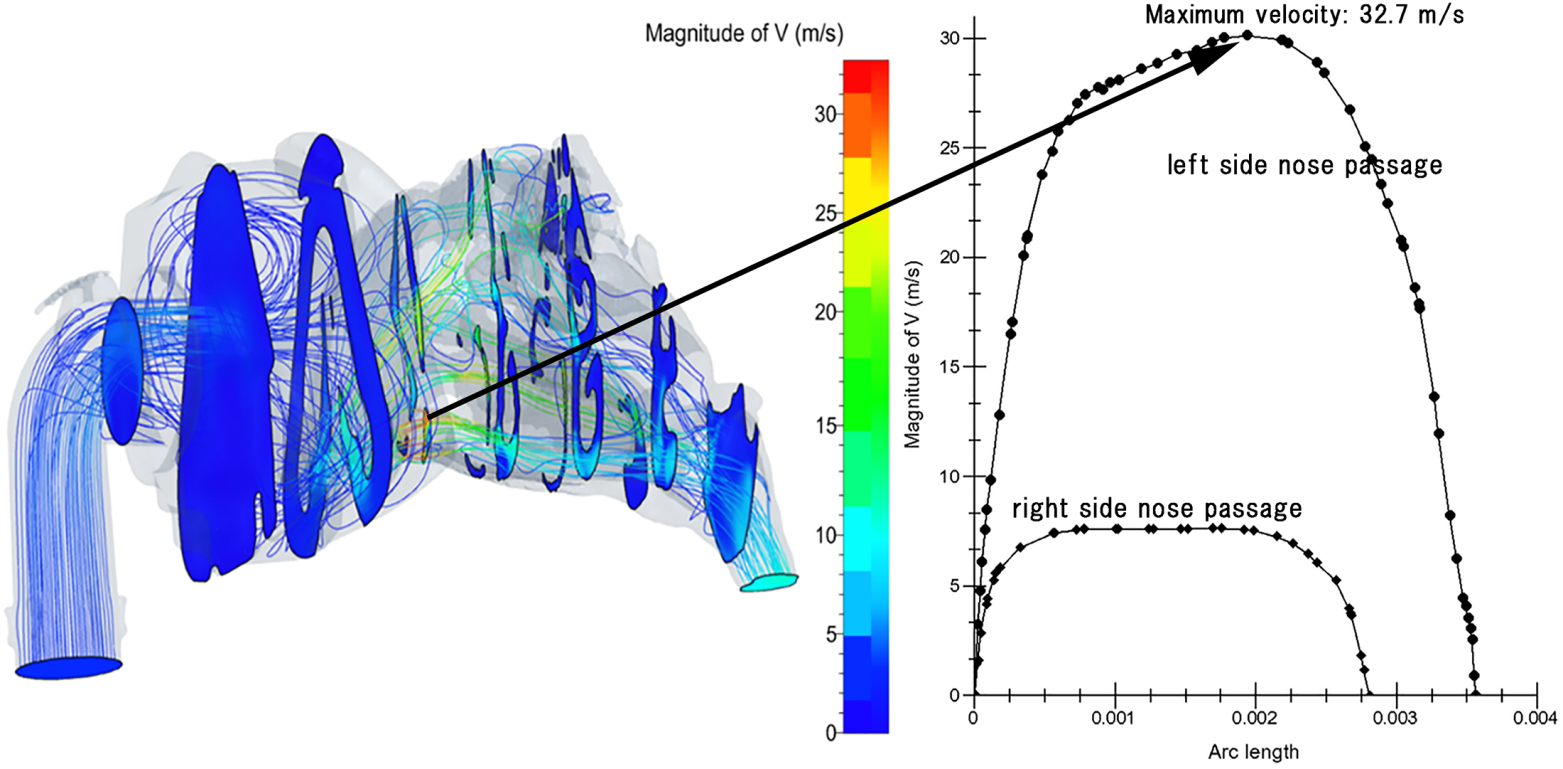
Airflow and velocity distributions in the nasal cavity and nasopharynx under 10 cmH_2_O CPAP (left panel). Velocity distributions in the nasal cavity and nasopharynx (right panel). Velocity contour mappings are shown in a colored distribution. Red streamlines indicate high velocity, and blue streamlines indicate low velocity, implying impaired airflow. Inspiratory airflow on the narrowed side became a rapid stream, as fast as 32.7 m/s. In contrast, the velocity on the normal side was 7.6 m/s. Airflow was not able to disperse across the entire cross-sectional area and thus became a concentrated, rapidly moving stream. Turbulence in front of the nostrils was seen. X-axis: distance from the nostrils; Y-axis: velocity (V, m/s) (right panel).

**Fig 5:**
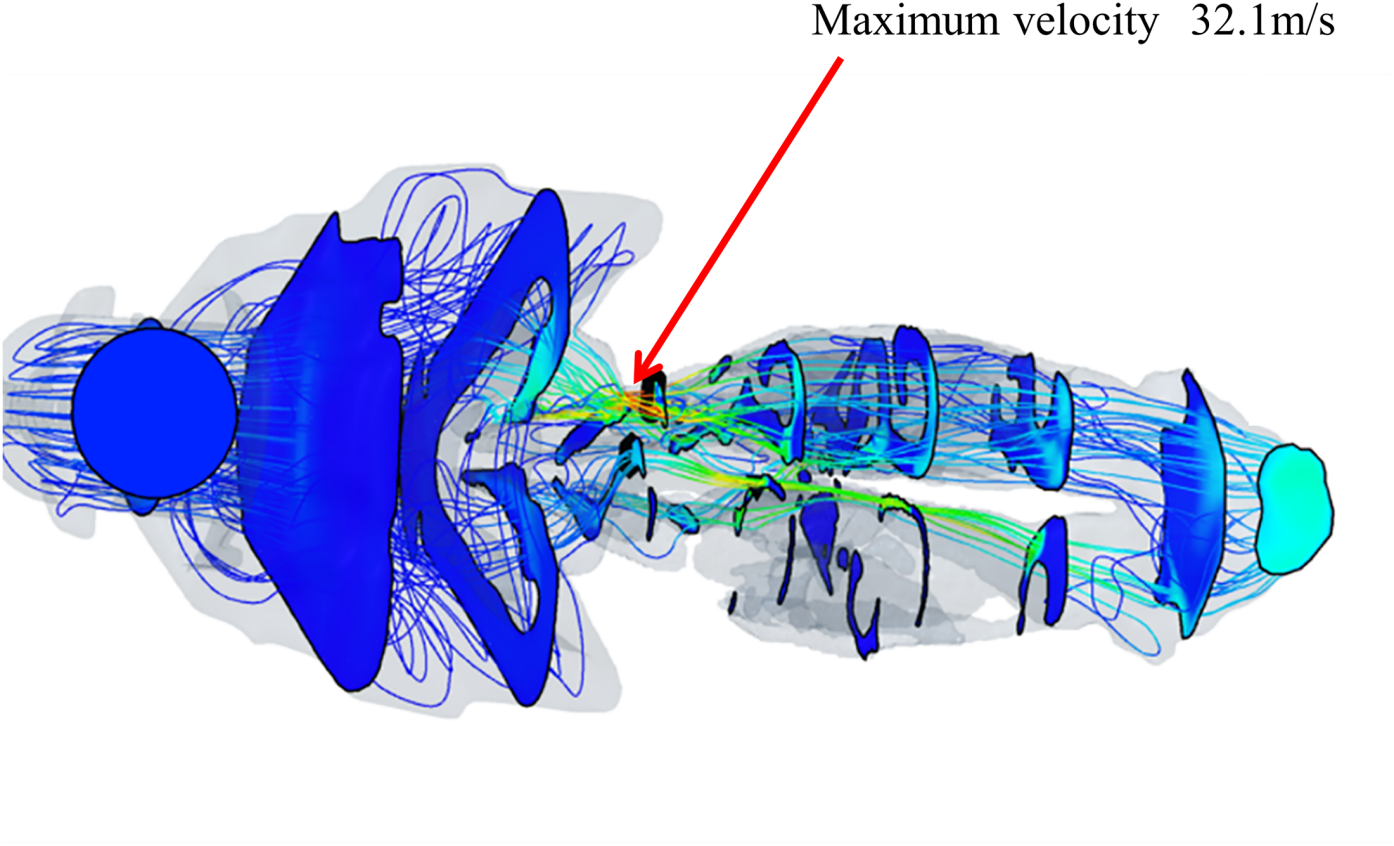
Caudal views comparing airflow and velocity contours with CPAP. Same patient as Fig 4.

**Fig 6:**
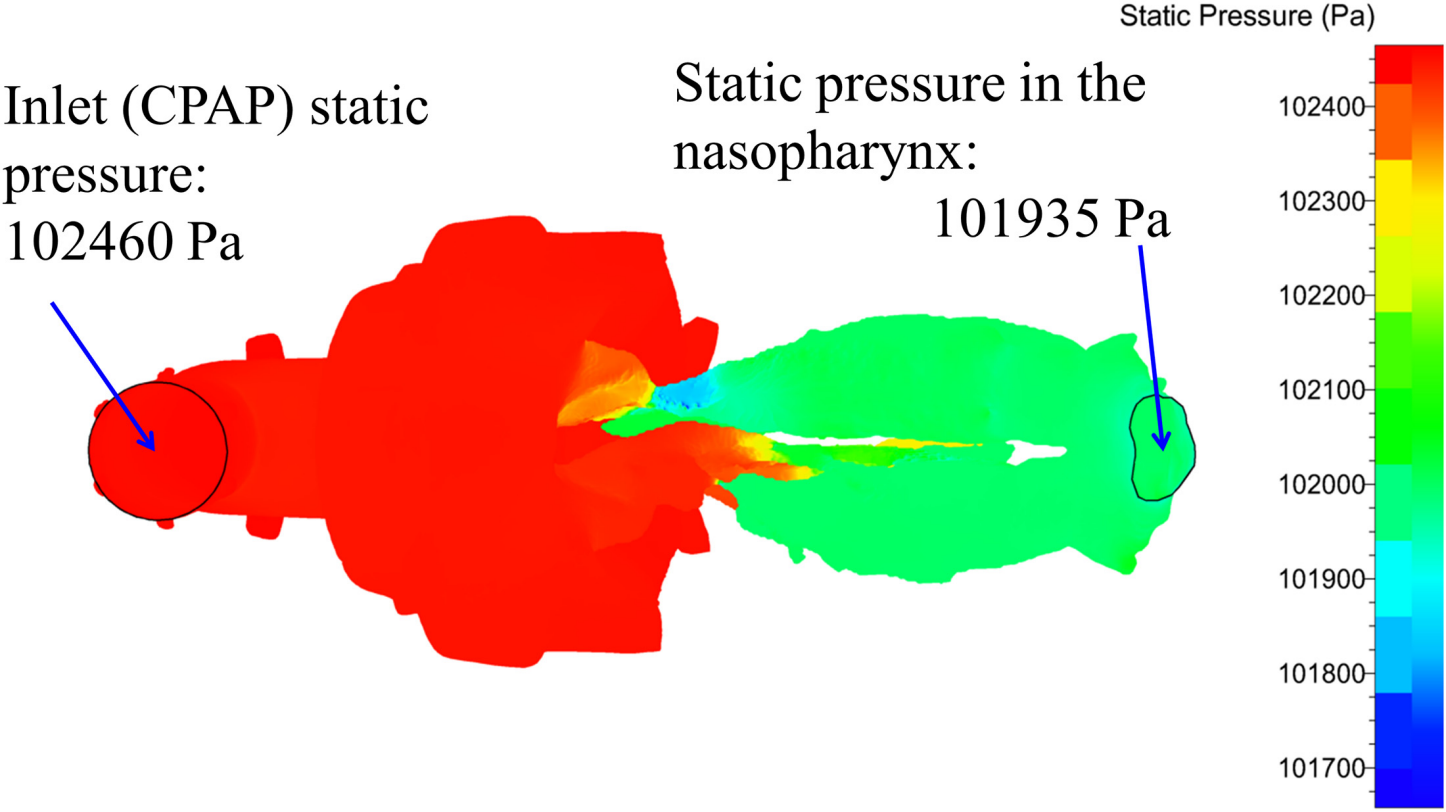
Inspiratory static pressure distribution (10 cmH_2_O, 889 ml/s). The pressure drop was 102460 − 101935 = 525 Pa = 5.35 cmH_2_O. Same patient as Fig 4.

Results of CFD simulations showed airflow streamlines and velocity contours in the nasal obstruction group. Typical results from individual subjects are shown in Figs 4–6 and group results are summarized in Table 1. There was a tendency of correlation between nasal resistance of CFD simulations without CPAP condition and that of actual measurements with rhinomanometry, however, it was not significant (Spearman ρ = 0.50, *p* = 0.391).

**Table 1:** Summary of CFD results under 10 cmH_2_O CPAP.

	Nasal obstruction group	Control group	*p*
Maximum velocity (m/s)	17.6 ± 7.6 (13.5−32.7)	11.8 ±1.8 (9.7−13.5)	*p* = 0.001
Inspiratory pressure drop (cmH_2_O) between mask and nasopharynx	2.44 ± 1.41 (1.22−5.35)	1.17 ± 0.29 (0.76−1.60)	*p* = 0.008
Inspiratory pressure loss coefficient (*Cp*)	18.9±9.2 (6.9−35.8)	8.3 ± 2.9 (5.3−14.5)	*p* = 0.012
Expiratory pressure in nasopharynx (cmH_2_O)	6.60 ± 0.19 (6.40−6.74)	6.47 ± 0.06 (6.39−6.58)	*p* = 0.11

Mean ± standard deviation (range).

Fig 4 shows the results of airflow and velocity contour mapping from CFD simulations in the nasal cavity and nasopharynx under 10 cmH_2_O CPAP and velocity distributions for the same patient. Caudal views of airflow and velocity contours for the same patient are shown in Fig 5. The narrowed (left) side showed impaired airflow and velocity contours. The airflow was not able to disperse across the entire cross-sectional area and thus became a concentrated, rapidly moving stream. Peak inspiratory airflow velocity on the left side reached 32.7 m/s, while that on the normal side was only 7.6 m/s. Turbulent flow occurred at relatively high local Reynolds numbers and was dominated by inertial forces. This turbulent flow tended to produce random vortices and chaotic flow fluctuations. Fig 6 shows the inspiratory static pressure distribution for the same patient shown in Figs 4 and 5. In the nasopharynx, the pressure decreased by 5.35 cmH_2_O relative to the inlet static pressure. In the control group, however, the mean decrease was only 1.17 ± 0.29 cmH_2_O.

Results are summarized in Table 1. There were significant differences between the nasal obstruction and control groups in maximum airflow velocity, inspiratory pressure drop between the mask and the nasopharynx, and inspiratory *Cp*. However, there was no significant difference in expiratory pressure in the nasopharynx between the groups.

**Fig 7:**
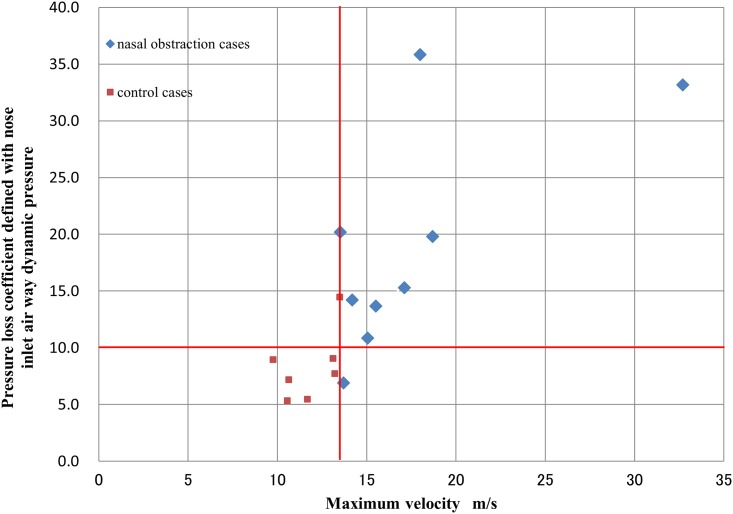
Scatter plot of maximum velocity versus inspiratory pressure loss coefficient (*Cp*). The nasal obstruction and control groups were separated by a velocity threshold of 13.5 m/s and *Cp* threshold of approximately 10.0. r = 0.776, *p*<0.001. Blue squares: patients with nasal obstruction; red squares: controls.

**Fig 8:**
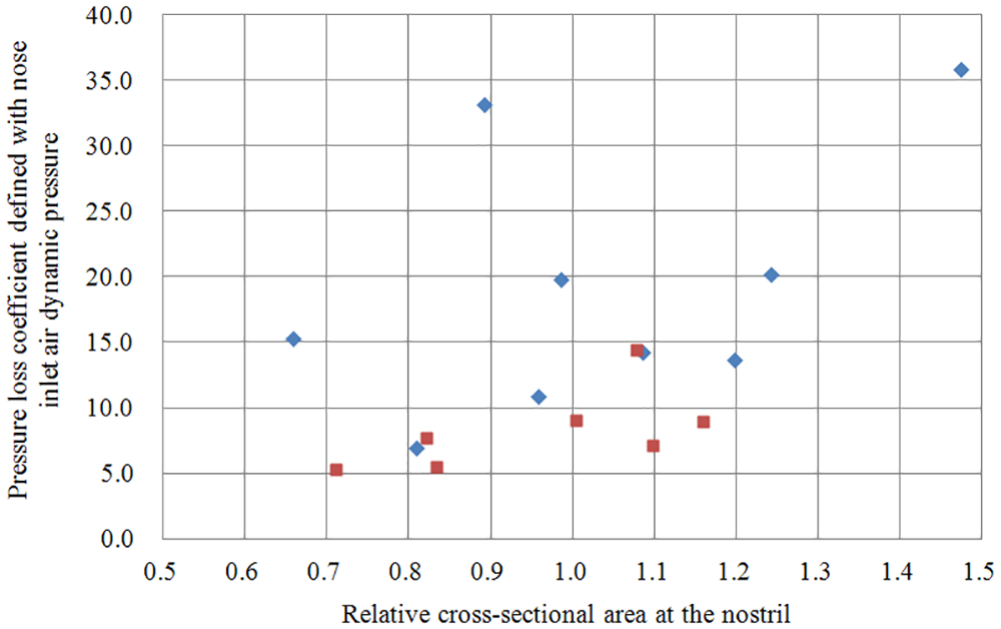
Scatter plot of relative cross-sectional area at the nostril versus inspiratory pressure loss coefficient (*Cp*). Note that the *Cp* thresholds were independent of the cross-sectional area at the nostrils. The longitudinal axis is the same *Cp* as Fig 7.

The scatter plot of *Cp* versus maximum airflow velocity (Fig 7) shows a strong correlation (r = 0.776, *p* < 0.001). The groups were separated by an airflow velocity threshold of precisely 13.5 m/s, and a *Cp* threshold of approximately 10.0. The *Cp* thresholds were independent of the cross-sectional area at the nostrils (Fig 8).

## Discussion

This is the first CFD analysis of the effect of nasal obstruction on CPAP treatment. Under 10 cmH_2_O CPAP, average peak inspiratory airflow on the narrowed side was nearly 50% higher in patients with nasal obstruction than in those without. Also, the average pressure drop in the nasopharynx in patients with nasal obstruction was more than double that in patients without (Table 1). There was significant difference in *Cp* between the nasal obstruction and control groups, whereas the *Cp* threshold was independent of the cross-sectional area at the nostrils (Fig 8).

The higher airflow velocity may activate mechanical or sensory receptors on the nasal mucosa responsible for sensing increased resistance to nasal airflow. When stimulation of the receptors exceeds a certain threshold, arousal or awakening would be triggered at the level of the central nerve system via trigeminal nerve input, resulting in sleep disturbance and discomfort. The end result may be CPAP intolerance.

Stuck et al. investigated cortically generated chemosensory event-related potentials during sleep and found that stimulation with a selective trigeminal irritant (CO_2_) produced a concentration-dependent increase in arousal frequency, suggesting that chemosensory stimulation is processed at the cortical level and elicits cortical activation during sleep [7,8]. Our previous study showed that nasal obstruction is predictive of spontaneous arousal-related oral flow during sleep; such oral flow begins during stable breathing and is preceded by spontaneous arousal but rarely accompanied by apnea or hypopnea [4]. The time fraction of this type of oral flow relative to total oral flow was significantly greater in patients with nasal obstruction than in those without [4].

The present study predicted an average pressure drop of 2.44 ± 0.44 cmH_2_O in the nasopharynx relative to inlet static pressure in the nasal obstruction group. When a transnasal pressure drop between the nostrils and the nasopharynx occurs, CPAP pressure needs to be increased, leading to greater patient discomfort.

The aerodynamics of nasal airflow are complex due to the geometry of the nasal cavity. However, CFD offers an accurate and highly graphical model to understand the nature of nasal airflow structure. Other CFD models have simulated turbinate enlargement and septal deviation. Lee et al. simulated turbinate enlargement by expanding the inferior turbinate and found significant malfunction of the nasal valve, resulting in increased total negative pressure in the nasal cavity during the inspiratory phase of breathing [9]. In addition, the velocity of nasal airflow was reduced significantly around the head of the turbinate compared to a normal healthy nose. Nasal airflow was redistributed toward the upper part of the nasal cavity, and the higher flow velocities increased wall shear stress at the olfactory zone. Wexler et al. suggested that the greatest reduction in pressure and regional mean air velocities occurred at the narrowest region around the inferior turbinate after virtual inferior turbinate reduction [10]. There was concomitant reduction in regional mean air velocities throughout the nasal cavity, especially in the posterior region. They also demonstrated that the airflow was redistributed inferiorly along the floor of the nasal cavity. Garcia showed that the airstreams flowed mainly through the upper half of the nasal cavity, while a low-velocity eddy occurred in the lower half in a patient with atrophic rhinitis [11]. CFD modeling of severe septal deviation revealed that the main inspiratory airstreams were channeled away from the common meatus toward the floor and roof of the nasal cavity at higher velocity, bypassing the area of deviated septum [12]. In addition, the area of high kinetic energy at the head of the inferior turbinate was lost, and high wall shear stress was noted in areas of high airflow velocity [13].

CFD assessments can also predict whether pediatric and adult patients might have OSA [14,15]. Furthermore, CFD assessments have demonstrated clear effects of nasal surgery and oral appliance treatment on upper airway airflow patterns [16,17]. The most promising application of CFD analyses may be to provide a personalized treatment plan for each patient, especially in cases where surgical treatments are considered.

There are several limitations to this study. First, although verification and validation of actual values at every point may be desirable for CFD analyses, actual measurements of airflow velocity in the nasal cavity and nasopharynx during sleep are not realistic. Second, only the nasal and nasopharyngeal passages were considered. It may also be important to consider other structures of the upper respiratory tract such as oropharyngeal and hypopharyngeal airways. Indeed, it has been speculated that the decreases in negative pressure in the nasopharynx due to reduced nasal airflow can adversely affect the function of the surrounding muscles and the mobility of the soft palate, which are common etiological factors for OSA. Third, we did not conduct advanced analysis of fluid-structural interactions, including elasticity, hardness, temperature, humidity, and flutter. Also unclear are the tolerable airflow velocity in the nasal cavity and the degree to which nasal resistance influences pharyngeal collapse. Further study of these issues is warranted.

## Conclusions

CFD analysis predicted that nasal obstruction under 10 cmH_2_O CPAP results in a rapid focal airstream in the nasal cavity with substantially higher peak velocity than predicted in the unobstructed nasal cavity, as well as a larger average pressure drop in the nasopharynx. A strong correlation between *Cp* and maximum airflow velocity was found. The nasal obstruction and control groups were separated by a velocity threshold of 13.5 m/s and a *Cp* threshold of approximately 10.0.

## Abbreviations

BMI: body mass index
CFD: computational fluid dynamics
*Cp*: pressure-loss coefficient
CPAP: continuous positive airway pressure
CT: Computed tomography
MRI: magnetic resonance imaging
OSA: obstructive sleep apnea
*Pin*: inlet pressure at the nostrils
*Pout*: outlet pressure at the nasopharynx
STL: stereolithography
*Vin*: air velocity at the nostrils
3D: three-dimensional
